# Clinical characterization of Collagen XII‐related disease caused by biallelic *COL12A1* variants

**DOI:** 10.1002/acn3.52225

**Published:** 2025-02-09

**Authors:** Riley M. McCarty, Dimah Saade, Pinki Munot, Chamindra G. Laverty, Hailey Pinz, Yaqun Zou, Meghan McAnally, Pomi Yun, Cuixia Tian, Ying Hu, Lucy Feng, Rahul Phadke, Sophia Ceulemans, Pilar Magoulas, Andrew J. Skalsky, Jennifer R. Friedman, Stephen R. Braddock, Sarah B. Neuhaus, Denise M. Malicki, Matthew N. Bainbridge, Shareef Nahas, David P. Dimmock, Stephen F. Kingsmore, Timothy E. Lotze, A. Reghan Foley, Francesco Muntoni, Volker Straub, Sandra Donkervoort, Carsten G. Bönnemann

**Affiliations:** ^1^ Neuromuscular and Neurogenetic Disorders of Childhood Section, National Institute of Neurological Disorders and Stroke National Institutes of Health Bethesda Maryland USA; ^2^ Department of Pediatrics, Roy J. and Lucille A. Carver College of Medicine University of Iowa Iowa City Iowa USA; ^3^ The Dubowitz Neuromuscular Centre Great Ormond Street Institute of Child Health London UK; ^4^ Department of Neurosciences University of California San Diego San Diego California USA; ^5^ Division of Medical Genetics Saint Louis University St. Louis Missouri USA; ^6^ Division of Neurology, Cincinnati Children's Hospital Medical Center & Department of Pediatrics University of Cincinnati Cincinnati Ohio USA; ^7^ Dubowitz Neuromuscular Centre, Division of Neuropathology UCL Queen Square Institute of Neurology Queen Square London UK; ^8^ Division of Genetics Rady Children's Hospital San Diego California USA; ^9^ Department of Genetics Texas Children's Hospital Houston Texas USA; ^10^ Division of Pediatric Rehabilitation Medicine Rady's Children's Hospital San Diego California USA; ^11^ Department of Orthopaedic Surgery University of San Diego San Diego California USA; ^12^ Rady Children's Institute of Genomic Medicine San Diego California USA; ^13^ Department of Pediatrics University of California San Diego San Diego California USA; ^14^ Department of Pathology Rady Children's Hospital San Diego California USA; ^15^ Division of Neurology and Developmental Neuroscience Baylor College of Medicine/Texas Children's Hospital Houston Texas USA; ^16^ NIHR Great Ormond Street Hospital Biomedical Research Centre London UK; ^17^ John Walton Muscular Dystrophy Research Centre Newcastle University and Newcastle Hospitals NHS Foundation Trust Newcastle upon Tyne UK

## Abstract

**Objective:**

While there have been several reports of patients with dominantly acting *COL12A1* variants, few cases of the more severe recessive Collagen XII‐related disorders have previously been documented.

**Methods:**

We present detailed clinical, immunocytochemical, and imaging data on eight additional patients from seven families with biallelic pathogenic variants in *COL12A1*.

**Results:**

All patients presented with a consistent constellation of congenital onset clinical features: hypotonia, dysmorphic features, most notably gingival hypertrophy, prominent distal joint hyperlaxity, with co‐occurring contractures of large joints, and variable muscle involvement, evident both clinically and on muscle imaging. Five patients presented with a severe congenital phenotype manifesting with profound weakness, significantly delayed or minimal attainment of motor milestones, respiratory insufficiency, and feeding difficulties. Three patients presented with mild‐to‐moderate muscle weakness and delayed milestones but were able to achieve independent ambulation. Patients were found to have biallelic loss‐of‐function *COL12A1* variants, except for one family (p.I1393Ffs*11/p.A1110D). Consistent with the variable clinical spectrum, in vitro immunocytochemistry analysis in fibroblasts ranged from complete absence of Collagen XII expression in a patient with severe disease, to a mild reduction in a patient with milder disease.

**Interpretation:**

Here we characterize the clinical presentation, muscle imaging, and dermal fibroblast immunostaining findings associated with biallelic variants in *COL12A1,* further establishing *COL12A1* as a recessive myopathic Ehlers–Danlos syndrome (mEDS) gene, and expanding the clinical spectrum to include a milder EDS phenotype.

## Introduction

Collagen XII (COLXII) is the largest member of the fibril‐associated collagen with interrupted triple helix family. COLXII is a homotrimer assembled from three COLXII alpha chains encoded by *COL12A1*. It is essential for collagen fibril organization by bridging Collagen I‐containing fibrils bound at the collagenous domain with various extracellular matrix (ECM) proteins including tenascin‐X.^1^, ^2^ In addition to regulating matrix formation, COLXII is thought to be important for modulating mechanical properties of collagen fibrils in connective tissues and bone.^1^, ^3^

Pathogenic variants in *COL12A1* have only recently been associated with disease in mice and humans.^4^, ^5^ Both recessive variants, as well as dominantly acting variants in *COL12A1* have been reported to cause myopathic Ehlers–Danlos syndrome (mEDS), an overlap disorder involving connective tissue and muscle.^5^, ^6^ In the initial report, patients with recessive biallelic loss‐of‐function (LoF) *COL12A1* variants manifested with a severe congenital‐onset disease characterized by generalized hypotonia, muscle weakness with minimal attainment of motor milestones, feeding and respiratory difficulties, and striking joint laxity with coexisting milder contractures of larger joints.^5^ Heterozygous dominantly acting *COL12A1* variants, typically glycine substitutions or in‐frame exon skipping variants, cause a dominant‐negative interference of the Gly‐X‐Y containing triple helical (TH) domain. Patients manifest with a milder phenotype of motor delay, mild proximal or distal weakness, and joint hyperlaxity with pes planus deformities of the feet. While additional patients with dominant acting *COL12A1* variants are increasingly being recognized,^4^, ^7^, ^8^, ^9^ patients with recessive biallelic variants remain rare. Recently, a single patient with a homozygous *COL12A1* splice site variant was identified, presenting with congenital onset hypotonia and weakness, delayed motor milestones, progressive scoliosis, who nevertheless reported no physical limitations at age 47 years.^10^ The overall clinical spectrum, disease manifestation, and genotype–phenotype correlation of recessive *COL12A1*‐related disease (*COL12A1*‐RD) remains poorly understood.

We report eight patients from seven families with biallelic pathogenic variants in *COL12A1* presenting with a consistent phenotype of myopathic EDS characterized by congenital onset hypotonia, joint hyperlaxity with concurrent joint contractures, variable degrees of muscle weakness, and involvement as evident on MRI. Taken together, this series expands the phenotypic and mutational spectrum associated with the emerging group of recessive *COL12A1*‐RD to now also include a milder phenotype.

## Methods

### Patient recruitment and sample collection

All patients were identified through their local neuromuscular or genetic provider and underwent detailed clinical examinations. Samples including DNA muscle and skin biopsies were collected based on standard procedures. For research studies, written informed consent and age‐appropriate assent were obtained from each participant. Ethical approval for human subject research studies was obtained from the following institutional review boards: National Institutes of Health (NIH), National Institute of Neurological Disorders and Stroke (NINDS) (Protocol 12‐N‐0095), Rady's Children's Hospital/UCSD (UCSD IRB# 172020, 170437), Health Research Authority, NRES Committee East of England – Hatfield (REC 13/EE/0398), and NRES Committee North East – Newcastle & North Tyneside 1 (reference 08/H0906/28). UK Regional Ethics Committee REC (reference 06/Q0406/33).

### Genetic analysis

Whole exome sequencing (WES), whole genome sequencing (WGS), or next‐generation sequencing‐based panel testing was performed in all patients. Details of the clinical and research‐based genetic testing methods are listed in Supplementary Table 1. Confirmation of variants and segregation testing was performed through Sanger Sequencing for all patients.

### Imaging studies

Conventional T1‐weighted spin echo and short tau inversion recovery (STIR) of the lower extremities on a 3.0‐T Achieva Philips MRI system were obtained for F1P1 and F5P5. Leg muscle MRI in F4P4 was performed using 1.5 T scanner according to standard protocols axial T1‐weighted and STIR sequences were acquired covering thighs and calves. For F7P8 standard T1‐weighted images were acquired on a 3.0‐T scanner (Philips Intera Achieva, Siemens TIM Trio). Muscle ultrasound images were obtained using a Siemens S2000 with a 15‐MHz linear probe and scored based on the Heckmatt Scale.^11^

### Fibroblast culture and immunocytochemistry analysis

Dermal fibroblasts isolated from skin biopsy were grown in Dulbecco's modified Eagle medium with 10% fetal bovine serum (Life Technologies) in 5% CO_2_ at 37°C. For immunofluorescence staining, cells were cultured in the presence of 50 μg/ml L‐ascorbic acid phosphate (Wako) until 5 days post‐confluent, fixed with 4% paraformaldehyde (PFA), incubated with primary antibodies at 4°C overnight [guinea pig anti‐collagen XII (KG76, generated by Dr. Koch^12^) and rabbit anti fibronectin antibody (Sigma Aldrich)], the antibody labeling was detected with secondary antibodies for 1 h at room temperature [Alex 568‐conjugated goat anti‐guinea pig IgG and Alexa 488‐conjugated goat anti‐rabbit IgG (Molecular Probes)]. Immunoblotting analysis of overnight conditioned culture media from confluent fibroblast cultures were performed using rabbit anti‐collagen XII (KR75, generated by Dr. Koch),^12^ rabbit anti fibronectin (F3648, Sigma Aldrich), and mouse anti‐tubulin antibody (T5168, Sigma Aldrich) followed with appropriated infrared fluorescent dye labeled secondary antibodies (LI‐COR Biosciences).

## Results

### Clinical findings

Detailed clinical information for all patients, (five males and three females) is summarized in Table 1 and Supplemental Results, with ages ranging from 15 months to 18 years at the time of the most recent examination. Limited clinical details for F7P8 were previously reported.^13^

**Table 1:** Clinical findings of patients with biallelic *COL12A1* variants.

Family/Patient	F1/P1	F2/P2	F3/P3	F4/P4	F5/P5	F5/P6	F6/P7	F7/P8
*COL12A1* Variants	c.5794+2T>A (*p*), c.5269C>T; p.R1757* (m)	c.8464C>T; p.R2822* (homozygous)	c.6340+1G>T (homozygous)	c.946_947insA; p.V316fs*6 (homozygous)	c.3329C>A; p.A1110D (*p*), c.4177del; p.I1393Ffs*11 (m)		c.5664+1G>A (*p*), c.6127delG; p.V2043* (m)	c.5230+1G>A (homozygous)
Sex/Age at visit	F/15 months	F/18 years	M/4 years	M/13 years	F/3 years	M/15 months	M/3 years	M/16 years
Ethnicity/consanguinity	European/No	Palestinian/Yes	Afghani/Yes	Pakistani/Yes	European/No		European/No	Pakistani/Yes
Onset	Congenital	Congenital	Congenital	Congenital	Congenital	Congenital	Congenital	Congenital
Presenting symptoms	Global weakness Arthrogryposis	Hypotonia Arthrogryposis DDH	Hypotonia Arthrogryposis DDH	Hypotonia Distal arthrogryposis DDH	Hypotonia DDH	Hypotonia Hip clicks at birth	Congenital hip dysplasia Talipes equinovarus	Hypotonia
Dysmorphic features								
Head	Dolichocephaly Low‐set ears	Dolichocephaly		Plagiocephaly			Plagiocephaly	
Neck	Mild webbing	Mild webbing						
Ocular	Blueish sclera Epicanthal folds				Bluish sclera	Blueish sclera		
Face	Myopathic	Long narrow face	Myopathic					Myopathic
Jaw	Micrognathia	Micrognathia	Micrognathia				Micrognathia	
Palate	High arched with deep midline ridge	High arched	High arched	High arched	High arched	High arched	High arched Severe prominence of lateral palatine ridges	
Dentition	Gingival hypertrophy Dental eruption cysts	Gingival hypertrophy Dental malocclusion	Gingival hypertrophy		Gingival hypertrophy	Gingival hypertrophy	Gingival hypertrophy Dental malocclusion	
Skeletal	Prominent calcanei	Prominent calcanei Pectus excavatum Short lower limbs			Prominent calcanei Pectus excavatum	Prominent calcanei	Gracile ribs Multiple fractures at birth	Prominent calcanei
Other	Bell‐shaped chest Sacral dimple	Soft skin Webbing Shallow palmar creases Mild thenar atrophy		Soft skin	Overlapping fingers Reduced LE muscle bulk		Mild axillary pterygia	
Maximum motor development	Poor head control Unable to sit without support	Sat with support at age 3 years	Limited head control	Able to run, jump, climb stairs	Ambulatory at age 15 months	Ambulatory at age 16 months	Cruised at 11 months, although unable to pull to stand	Never achieved independent ambulation
Pattern of weakness (MRC)	UEs (3/5) LEs (2/5)	UEs and LEs (4/5) Neck flexion and deltoid (2/5)	Generalized weakness	Mild proximal and axial weakness	UEs and LEs (4+/5) Neck flexion (2/5)	UEs and LEs (4+/5) Neck flexion (2/5)	Proximal upper and lower extremities at least (3/5)	Distal > proximal weakness; LE > UE weakness
Joints								
Hyperlaxity	Distal and proximal	Distal and proximal	Distal	Distal hands and knees	Distal and proximal	Distal and proximal	Distal hands	Distal and proximal
Contractures	Long finger flexors Fingers Toes	Long finger flexors Knees	Fingers	Fingers	Hip adductors	Hip adductors	Fingers	Knees
Spine	Thoracic kyphoscoliosis	Progressive scoliosis	Kyphosis		Hyperlordosis		Scoliosis	Scoliosis
Cardiac involvement	Thickened mitral valve leaflets Mild‐moderate MR	HCM MVP WPW syndrome s/p ablation	Dysplastic mitral and tricuspid valve with redundant tissue Mild MR		Trivial mitral regurgitation			
Respiratory insufficiency	No	Yes	Yes	Yes	No	No	Yes	Yes
Neonatal feeding difficulties	Yes	Yes	Yes	Yes	Yes	No	Yes	Yes

DDH, developmental dysplasia of hip; EF, ejection fraction; F, female; FVC, forced vital capacity; HCM, hypertrophic cardiomyopathy; LEs, lower extremities; M, male; m, maternal; MR, mitral regurgitation; MRC, Medical Research Council; MVP, mitral valve prolapse; p, paternal; s/p, status post; UEs, upper extremities; WPW syndrome, Wolff–Parkinson–White syndrome.

**Figure 1:**
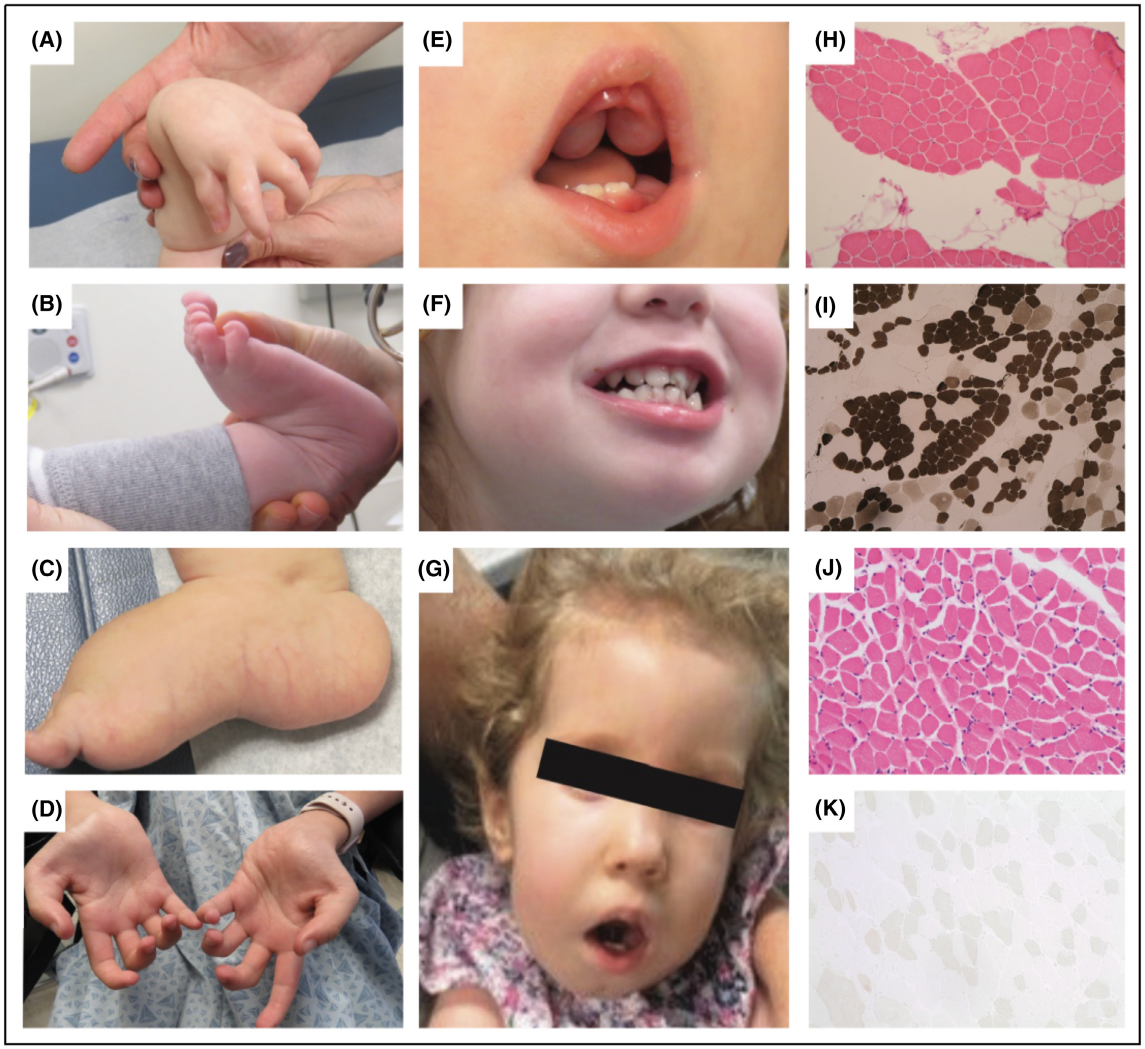
Common clinical and histological findings in patients with biallelic *COL12A1* variants. (A) Wrist hyperlaxity; finger, and long finger flexor contractures (F1P1). (B) Hyperlaxity of the ankle (F5P6). (C) Prominent calcanei (F1P1). (D) Long finger flexor contractures (F2P2). (E) High‐arched palate with a deep midline groove and dental eruption cysts (F1P1). (F) Gingival hypertrophy (F5P5). (G) Narrow elongated face with myopathic facies and open mouth posture (F1P1). (H) Biopsy of the vastus lateralis from F7P8 at age 8 months revealed mild fiber size variation with both hypertrophied, rounded and polygonal atrophic fibers on hematoxylin and eosin (H&E) with (I) mild Type 1 fiber predominance and Type 1 hypotrophy on ATPase pH 4.6. (J) Biopsy of the vastus lateralis from F2P2 at age 7 years revealed very subtle variation in fiber size without clear predominance of Type 1 fibers on ATPase pH 4.3 (K).

All patients presented with congenital onset symptoms including reduced fetal movement, hypotonia, and hip dysplasia, with four patients (F1P1, F2P2, F3P3 and F4P4) presenting with arthrogryposis. Patient F6P7 was found to have multiple (left femur fracture and left tibia) fractures at birth. Neonatal feeding difficulties were present in seven, requiring a nasogastric or gastrostomy tube placement. Respiratory involvement was variable, ranging from none to tracheostomy and ventilatory dependency since birth (F6P7). All patients had delayed acquisition of motor milestones. Five patients presented with a severe congenital phenotype manifesting with profound weakness and joint hypermobility, minimal attainment of milestones accompanied by respiratory failure and feeding difficulties. Three patients (F4P4, F5P5, and F5P6) presented with milder disease with mild to moderate muscle weakness and were able to achieve independent ambulation at age 3 years, age 15 months, and age 16 months, respectively. None of the patients had significant worsening of motor symptoms, instead showing steady improvement over time. F2P2, F6P7 and F7P8 had progressive scoliosis requiring spinal surgery at age 10 years in F2P2. F1P1 and F3P3 were noted to have progressive thoracic kyphoscoliosis. Distal joint laxity was noted in all patients (Fig. 1A–C), typically affecting wrists and ankles. Concurrent joint contractures were present in six patients and typically affected the long finger flexors (Fig. 1D) as well as the knees.

Dysmorphic features were evident in all patients. Patients F1P1 and F2P2 were found to have dolichocephaly, and F4P4 and F6P7 presented with plagiocephaly. Other common findings included micrognathia (*n* = 4), gingival hypertrophy (*n* = 6) (Fig. 1E), dental abnormalities (*n* = 3) (Fig. 1F), bluish hue to the sclerae (*n* = 3), myopathic facies (*n* = 4) (Fig. 1G), and webbing of joints (*n* = 2). Four patients also had evidence of cardiac involvement, with one (F2P2) presenting with Wolff–Parkinson–White syndrome. F1P1 and F3P3 had echocardiograms in infancy showing mild valvular anomalies with thickened mitral valve leaflets and mild‐to‐moderate mitral regurgitation in F1P1 and dysplastic mitral and tricuspid valves with redundant tissue and mild mitral valve regurgitation in F3P3. Creatine kinase (CK) levels were normal in F1P1, F3P3, F5P5, and F5P6, but mildly elevated in F2P2 and F6P7 (500 U/L range). Muscle biopsy in F2P2, F3P3, F4P4, and F7P8 revealed subtle variation in fiber size, consistent with a mild myopathic process, with internalized nuclei seen in F3P3 (Fig. 1H–K).

Taken together, patients presented with a constellation of symptoms including congenital onset hypotonia, dysmorphic features with notable gingival hypertrophy, prominent distal joint hyperlaxity with concurrent contractures of large joints and variable degrees of muscle involvement, cardiac involvement, respiratory insufficiency, and feeding difficulties.

### Muscle imaging

**Figure 2:**
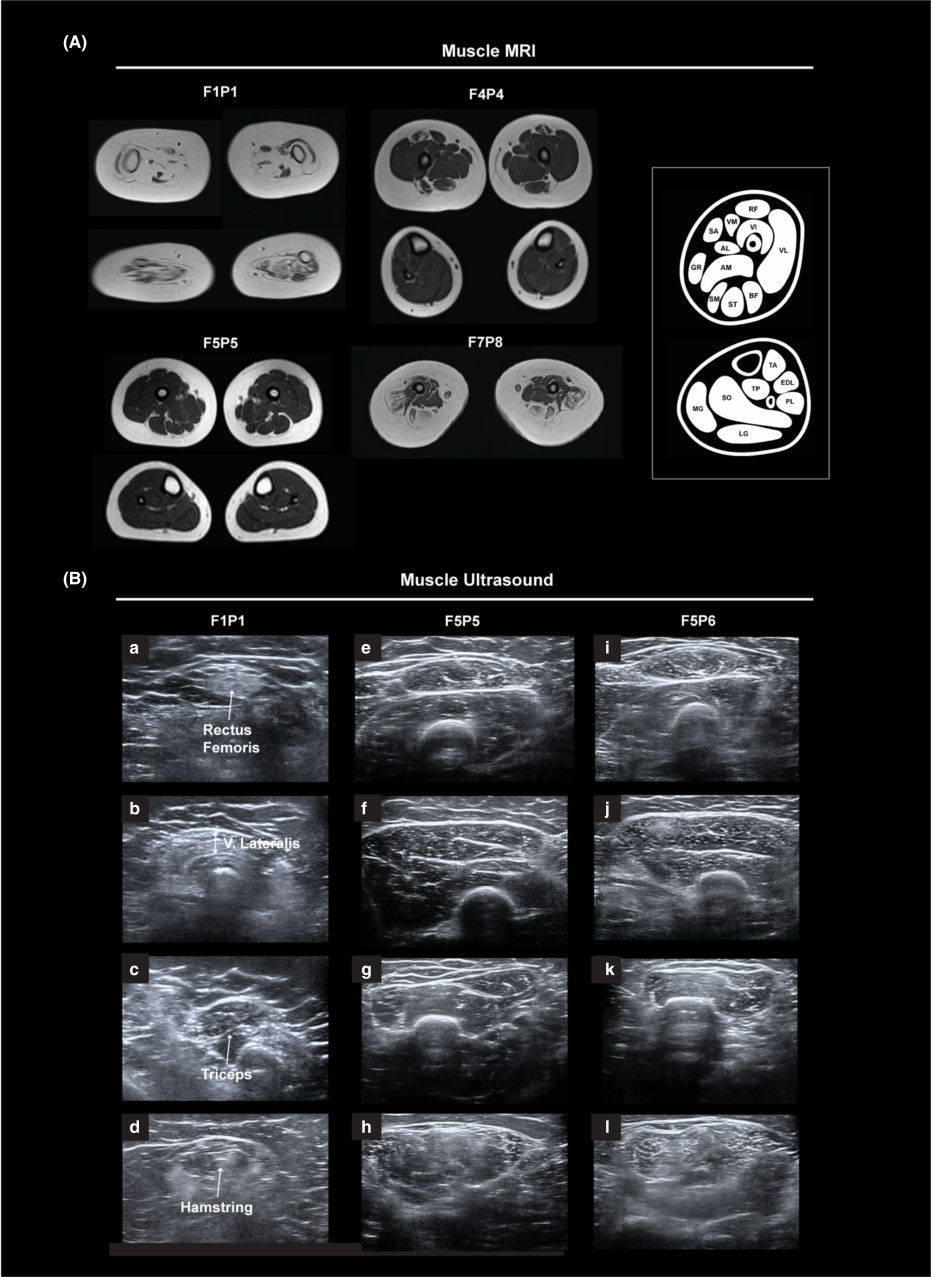
Muscle imaging in patients with biallelic *COL12A1* variants. (A) Muscle MRI, axial T1 at the thigh (top) and lower leg (bottom) for F1P1 (left, top) and F4P4 (middle, top), at age 15 months and 11 years, respectively. There was severe generalized muscle atrophy and fatty replacement in F1P1's proximal and distal lower extremity. Asymmetry and imaging artifact in the lower leg was due to contractures. F4P4's MRI demonstrated significant posterior thigh involvement with relative sparing of the semitendinosus. Preserved medial and anterior compartments of the thigh, with the exception of the rectus femoris with atrophy and fatty infiltrates noted around the central fascia. Muscle MRI in F5P5 (left, bottom) at age 6 years demonstrating normal appearance of muscles of the thigh and distal lower extremity. Muscle MRI of F7P8 (right, bottom) at age 7 years revealed relative sparing of the medial and anterior thigh compartments compared to the posterior thigh compartment, with selective atrophy of the rectus femoris, and to a lesser extent the vastus lateralis. Visualization of normal muscle imaging (far right). (B) Muscle ultrasound of F1P1 (age 15 months), F5P5 (age 6 years), and F5P6 (age 4 years) of the rectus femoris (RF) (a, e, and i), vastus lateralis (V. Lateralis) (b, f, and j), triceps (c, g, and k), and hamstring (d, h, and l). Imaging in P1 demonstrated diffuse muscle atrophy in the lower extremity (LE) more than upper extremity (UE) with dense granular increased echogenicity in the rectus femoris and vastus lateralis, and mixed pattern echogenicity in the triceps. Muscle ultrasound in F5P5 and F5P6 revealed mild–moderate granular/streaky echogenicity, most pronounced in the hamstrings and rectus femoris with no muscle atrophy.

Muscle MRI was available for F1P1 (age 15 months), F4P4 (age 11 years), F5P5 (age 6 years), and F7P8 (age 7 years) (Fig. 2A). F1P1 had evidence of diffuse severe muscle atrophy with fatty replacement throughout the proximal and distal lower extremity muscles bilaterally. Some asymmetry and imaging artifact related to contractures was noted in the lower leg. In contrast, MRI in F4P4 revealed mainly posterior thigh involvement with relative sparing of the semitendinosus muscle. The medial and anterior compartments of the upper leg were relatively preserved, aside from the rectus femoris, which showed mild atrophy and fatty infiltrates around the central fascia. Muscles of the distal lower extremity were normal. MRI in F5P5 was normal, demonstrating normal appearance of muscles of the thigh and distal lower extremity. MRI in F7P8 revealed relative sparing of the anterior and medial thigh compartments compared to the posterior thigh. Within the anterior thigh, the rectus femoris was most involved, with mild atrophy and some instances of fatty infiltrates along the central fascia. A lesser degree of atrophy and fatty infiltration of the vastus lateralis was seen.

Muscle ultrasound images were obtained for F1P1 (age 15 months), F5P5 (age 6 years), and F5P6 (age 4 years) (Fig. 2B). Muscle ultrasound in F1P1 revealed diffuse muscle atrophy (lower extremities more than upper extremities) with increased echogenicity with a dense, granular appearance in the rectus femoris and vastus lateralis. Increased echogenicity with a mixed granular/streaky pattern was present in the triceps. Muscle ultrasound in F5P5 and F5P6 revealed diffuse mild granular and streaky increased echogenicity with greater posterior thigh involvement, relative sparing of the medial and anterior compartments of the upper leg, and relatively preserved appearance of the distal lower extremity muscles. In the upper extremity, increased echogenicity with a mixed granular/streaky pattern was present in the triceps. There was no muscle atrophy.

### Identification of *COL12A1* variants

Disease occurrence was sporadic in six families, affecting only a single family member. Family history was significant in family (F5), with two affected siblings born to unaffected parents. Four families (F2, F3, F4, and F7) reported consanguinity. The sister of F4P4 was diagnosed with congenital myotonia caused by a homozygous pathogenic variant in *CLCN1* (c.1696G>A p.(Ala566Thr)). F4P4 was found to be heterozygous for this variant.

**Figure 3:**
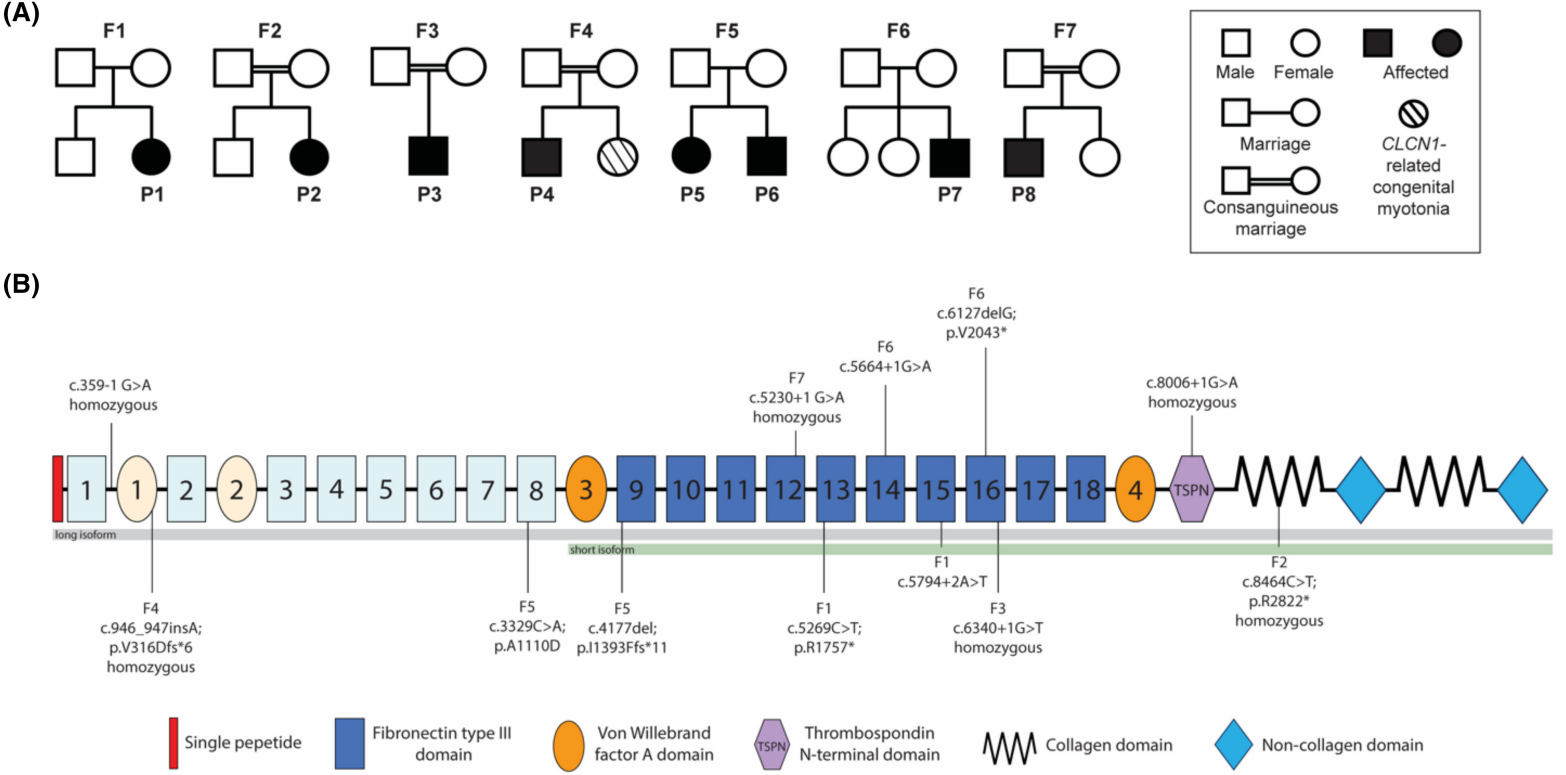
Pedigrees and genetic findings in patients with biallelic *COL12A1* variants. (A) Pedigrees of the families with biallelic *COL12A1* variants. (B) Collagen XII α1 domains and structure with labeled recessive variants: *COL12A1* recessive variants described in this paper (lower) and those reported previously (upper) were labeled on Collagen XII α1. The short isoform is depicted in bright colors; the additional domains in the long splice isoform are added as lighter symbols.

Family history was otherwise non‐contributory (Fig. 3A). Next‐generation‐based sequencing identified rare biallelic *COL12A1* (NM_004370.6) variants in all eight patients. Details of the *COL12A1* variants can be found in Figure 3B and in Table S1. Four patients (F2P2, F3P3, F4P4, and F7P8) were found to be homozygous for either a splice site or a truncating variant, and two patients (F1P1 and F6P7) were compound heterozygous for different LoF alleles. Variants were either rare, or absent, from gnomAD v4.0.0.^14^ *COL12A1* is constrained for LoF variation, with only 102 of 320.4 expected predicted LoF variants observed in gnomAD v4.0.0 (pLI score 0.97, LOEUF score 0.38^14^).

The two affected siblings (F5P5 and F5P6) in Family 5 were found to be compound heterozygous for a maternally inherited LoF allele c.4177del; p.I1393Ffs*11 and a paternally inherited c.3329C>A; p.A1110D missense variant. The missense variant is absent from gnomAD v4, and is highly conserved. There are three different variants at the same residue p.A1110T (listed once), p.A1110S (listed three times), and p.A111V (listed twice). The p.A1110D *COL12A1* variant has inconsistent in silico computational predictions (SIFT: deleterious, polyphen‐2: probably damaging) and has not yet been reported to cause disease.

Five patients (F1P1, F2P2, F3P3, F6P7, and F7P8) were found to have either homozygous or compound heterozygous LoF variants impacting both the long (NM_004370.6) and the short isoform (NM_080645.3) (Fig. 3B). F4P4 was homozygous for a LoF variant, which impacted the long isoform only, leaving the short isoform intact. F5P5 and F5P6 (Family 5) were compound heterozygous for a paternal missense variant (p.A1110D), which impact the long isoform and leaves the short isoform intact, and a maternal LoF variant that impacts both isoforms.

### Immunocytochemistry

**Figure 4:**
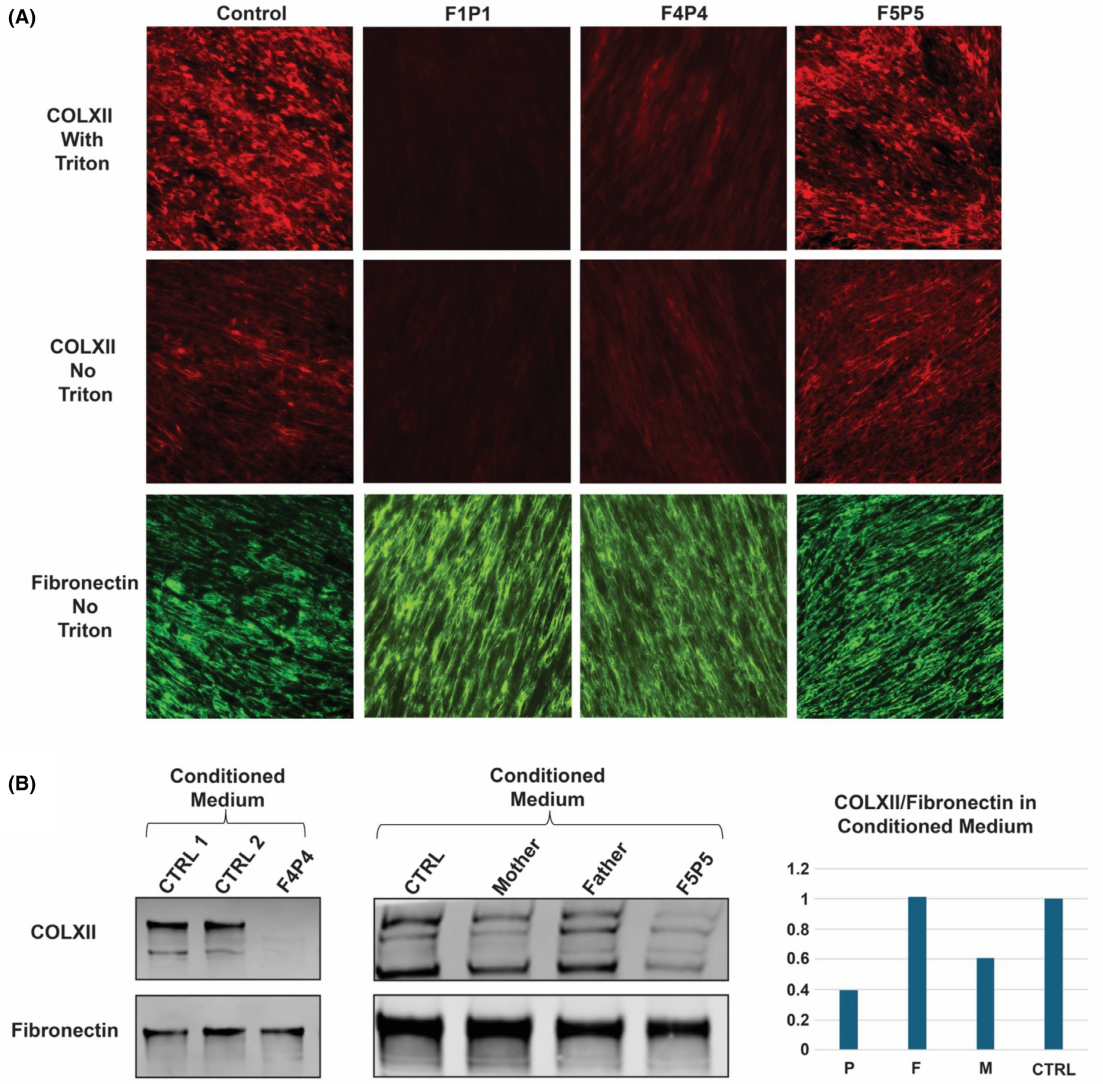
Collagen XII expression in cultured fibroblasts. (A) Immunofluorescence (IF) analysis of Collagen XII ECM formed in 5‐day culture, compared with normal control fibroblasts, F1P1 fibroblasts showed no detectable intracellular or extracellular immunoreactivity for Collagen XII; the signal of Collagen XII from F4P4 fibroblasts was detectable but significantly reduced while the signal from F5P5 is similar compared to normal control. (B) Immunoblotting of overnight conditioned medium of the fibroblast cultures with quantification. Compared to normal control fibroblasts, Collagen XII signal was barely detectable in F4P4 fibroblasts; was reduced in F5P5, but normal in both heterozygous unaffected carrier parents (P: p.I1393Ffs*11 / p.A1110D, F: p.A1110D, M: p.I1393Ffs*11) . P = patient; F = father; M = mother. Fibronectin was used as staining/loading control.

Immunofluorescence staining of fibroblasts was performed in a control and in three patients (F1P1, F4P4, and F5P5) (Fig. 4A). COLXII immunocytochemistry in control fibroblasts revealed both intracellular (Triton X100 treated culture) and extracellular (ECM) Collagen XII immunoreactivity. In comparison, no detectable intracellular or extracellular Collagen XII immunoreactivity was seen in F1P1 (c.5794+2T>A/p.R1757*), while F4P4 (homozygous p.V316fs*6) showed a reduced intracellular and extracellular Collagen XII immunoreactivity. In F5P5 (p.I1393Ffs*11/ p.A1110D), no significant Collagen XII abnormality was observed in the fibroblast cultures compared to control.

In addition, we analyzed the secreted soluble Collagen XII expression in the overnight conditioned medium of the fibroblast cultures by immunoblotting for F4P4 and F5P5 (Fig. 4B). In F4P4, the secreted soluble Collagen XII signal was barely detectable, while it was reduced in F5P5 compared to both heterozygous unaffected parents and a control sample.

## Discussion

Both recessive and dominant mutational mechanisms in *COL12A1* cause an emerging overlap syndrome involving muscle and connective tissue. While additional dominant cases have recently been reported, there have been fewer cases of the originally more severe recessive *COL12A1*‐RD identified. In this series, we provide detailed clinical, imaging, and immunocytochemical data for eight patients from seven families with biallelic variants in *COL12A1*. All patients manifest a consistent phenotype of congenital onset hypotonia, dysmorphic features, including most notably gingival hypertrophy, prominent widespread but distally more pronounced joint laxity with variable concurrent joint contractures, and variable degrees of muscle weakness, cardiac involvement, respiratory insufficiency, and feeding difficulties. Muscle involvement ranged from profound weakness with significantly delayed or minimal attainment of motor milestones, to isolated hip flexor weakness without functional limitation. Five patients presented with a severe congenital phenotype, while three patients (F4P4, F5P5, and F5P6) presented with milder disease, and were able to achieve independent ambulation. All patients demonstrated slow steady improvements in motor function over time without clinical regression.

The clinical diagnosis of EDS is based on the presence of clinical hallmarks of EDS including joint hypermobility, skin hyperextensibility, and tissue fragility, with further delineation of the specific EDS subtypes relying on characteristic phenotypic findings such as neuromuscular manifestations, with subsequent confirmatory genetic testing.^15^ Within the EDS spectrum, there can be substantial overlap with primary EDS, those with myopathic or kyphoscoliotic EDS, and myopathies with connective tissue involvement.^16^ *COL12A1‐*RD, emerges as a clinical overlap syndrome involving muscle and connective tissue, alternatively also classified as myopathic EDS. The spectrum of underlying neuromuscular manifestations of *COL12A1*‐RD remain insufficiently characterized given the limited number of patients identified and reported thus far, in particular for the more severe recessive form of the disease, which, based on the number of identified patients, appears to be extremely rare. These patients present with congenital hypotonia and often initially profound, albeit nonprogressive, muscle weakness, but in time may improve somewhat from a functional motor standpoint. While clinical aspects of a hereditary disorder of connective tissues are prominent, the changes identified on muscle imaging in combination with muscle biopsy in four patients demonstrating mild myopathic findings without dystrophic features support a concomitant myopathic process.

While clinically distinct, findings of distal joint laxity with coexisting contractures in the setting of muscle weakness in patients with *COL12A1*‐RD, partially resemble patients with Collagen VI‐related dystrophy (COL6‐RD).^17^ COL6‐RD encompasses a spectrum of disease ranging from the severe Ullrich congenital muscular dystrophy, to intermediate and Bethlem myopathy on the milder end, and is therefore a relevant consideration in the differential diagnosis of the *COL12A1*‐RD spectrum. However, the current OMIM classification for *COL12A1* that includes Ullrich congenital muscular dystrophy 2 and Bethlem myopathy (OMIM: 120320) should be reconsidered as there are distinct phenotypic differences. The Collagen XII disease spectrum might be better viewed as a form of myopathic EDS. In fact, this is the designation applied in the latest EDS nomenclature.^15^, ^18^

Patients with COL6‐RD typically present with progressive muscle weakness and joint contractures, and also have distally predominant joint laxity, which is less widespread than is seen in *COL12A1*‐RD where it also affects some of the larger joints. Therefore, striking more widely distributed joint laxity, less prominent contractures, and nonprogressive muscle weakness allowing for some clinical improvement of motor function (in particular in patients with less severe involvement at the outset), are important features to help distinguish patients with *COL12A1* myopathic EDS from those with COL6‐RD in whom the muscle involvement can be quite progressive and dystrophic in nature, including progressive respiratory failure. In addition, specific oral phenotypic features, namely gingival and dental abnormalities are atypical for COL6‐RD. Interestingly, while both disease groups present with velvety skin of palms and feet, patients in this series do not display other skin findings including keratosis pilaris and keloid scarring which are often seen in COL6‐RD.^19^ Early kyphosis and progressive scoliosis are a common finding in *COL12A1*‐RD^5^, ^10^ and can also be seen in COL6‐RD as well as other forms of kyphoscoliotic EDS including *FKBP14* and *PLOD1*‐related disease, with hearing loss being an important differential seen in *FKBP14*‐related disease.^20^, ^21^

Muscle imaging can serve as useful noninvasive diagnostic tool to aid in the clinical differential diagnosis, with COL6‐RD often characterized by increased signal along fascial planes and, giving rise to a so‐called “central cloud” along the central fascia of the rectus femoris, and a characteristic rimmed appearance of the vastus lateralis. This typical COL6‐RD‐associated pattern is different from our observations in patients with *COL12A1*‐RD, in whom a different pattern of muscle involvement may be emerging. Among the three patients presenting with milder disease (F4P4, F5P5, and F5P6), the hamstrings appear most affected, with some involvement of the rectus femoris, and lesser involvement of the vastus lateralis. While imaging reveals diffuse muscle atrophy and fatty replacement in severely affected F1P1, imaging of F7P8 in comparison with the milder patients confirms the predominant hamstring, and lesser anterior thigh muscle pattern of involvement. Interestingly, the relative more prominent involvement of the rectus femoris among the muscles of the quadriceps has also been noted in an older patient with dominant *COL12A1*‐RD and in patients with *FKBP14*‐RD as another myomatrix disease.^6^, ^20^

In addition to the initially reported severe congenital onset phenotype associated with biallelic null variants in *COL12A1*, this series of eight additional patients both confirms the severe end, but expands the clinical spectrum, to now include a milder connective tissue muscle overlap phenotype. This milder phenotype also presents with a congenital onset, but demonstrates subsequent steady improvement in motor function allowing for the acquisition of milestones such as independent ambulation. All five (P1, P2, P3, P7, and P8) patients presenting with severe disease had biallelic LoF variants affecting both the long (NM_004370.6) and the short (NM_080645.3) isoforms (Fig. 3B). These variants are expected to cause nonsense‐mediated mRNA decay (NMD) of both isoforms, consistent with the absent COL12 IF signal as shown in P1's fibroblast culture. The homozygous truncating variant (p.V316Dfs*6) in P4 is expected to cause NMD of the long isoform only and leaves the short isoform intact, resulting in a significantly reduced COL12 signal in both IF and conditioned medium. This result also implies that long isoform might be the major expression pattern of dermal fibroblasts in culture condition.

Recently, a homozygous *COL12A1* splice variant was identified in a patient with congenital hypotonia, weakness, joint laxity, and progressive scoliosis who was able to achieve independent ambulation and who did not report physical limitations in childhood.^10^ In our series we report three patients (P4, P5, and P6) with biallelic *COL12A1* variants who present with a similar milder disease, further establishing *COL12A1*‐related myopathy and expanding the phenotypic spectrum. The previously reported patient, in addition to the patients with milder clinical phenotypes in our series (F4P4, F5P5, and F5P6), had either one, or both variants impacting the *COL12A1* long isoform (Collagen XIIA1). In F5, the maternal variant (p.I1393Ffs*11) is expected to cause NMD of both the short and long isoform; while the paternal missense variant (p.A1110D) is expected to produce a mutant long isoform and intact short isoform. As a result, only a mildly reduced COL12 expression in the overnight conditioned culture medium was observed. In the extended culture condition, such as the deposited ECM for 5 days, however, the COL12 IF signal is similar to the normal control, but its function may be affected due to the substitution.

In mice, the long isoform is predominately expressed in early prenatal development, while postnatally it is mostly restricted to dense connective tissues, with the short isoform becoming more predominantly expressed.^22^, ^23^, ^24^ In chicken, however, it has been shown that the embryonic expression of the long isoform is more restricted in tissue compared to the short isoform.^23^ Taken together, the congenital presentation of patients with biallelic *COL12A1* variants with subsequent clinical improvement, albeit variable, perhaps points toward a previously underrecognized developmental function for both isoforms. In addition, we can see a genotype–phenotype correlation trend emerging with homozygous LoF variants impacting the short isoform having a more severe congenital phenotype with profound weakness, respiratory insufficiency and feeding difficulties and minimal attainment of motor milestones compared to patients with variants impacting the long isoform (Fig. 3B).

This series characterizes the clinical presentation, immunostaining, and muscle imaging findings associated with recessive biallelic variants in *COL12A1* and further establishes *COL12A1* myopathic EDS as a connective tissue/muscle overlap syndrome, with an expanding clinical spectrum from severe to milder phenotypes. Identification of additional variants in *COL12A1* and the characterization of the spectrum of associated clinical, histological, and imaging findings, specifically in older patients, will lead to further insight in the disease manifestations and emerging genotype–phenotype correlations associated with pathogenic biallelic *COL12A1* variants.

## Author Contributions

Study concept, design, and supervision: SD and CGB. Data acquisition: DS, PM, CGL, HP, YZ, MM, CT, YH, LF, RP, SC, PM, AJS, JRF, SRB, SN, DMM, MNN, SN, DPD, SFK, TEL, ARF, and VS. Data analysis and interpretation: RMM, PY, FM, and VS. Drafting the manuscript: SD, RMM, and DS. Critical review of the manuscript: All authors.

## Conflict of Interest

The authors have no conflict of interest to report.

## Supporting information


Figure S1.

Appendix S1.

## Data Availability

Data available on request from the authors.
