# Supplementary figures and images for: Clinical characterization of Collagen XII‐related disease caused by biallelic COL12A1 variants
Source: Ann Clin Transl Neurol. 2025 Feb 9;12(3):602–14. doi: 10.1002/acn3.52225 (PMC11920742; doi:10.1002/acn3.52225)

## Supplemental Figure

**A**

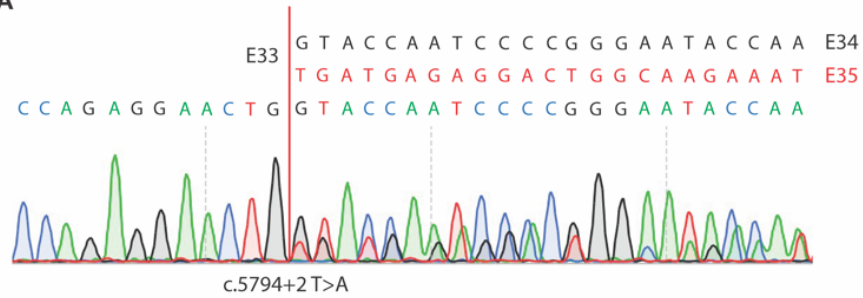

**B**

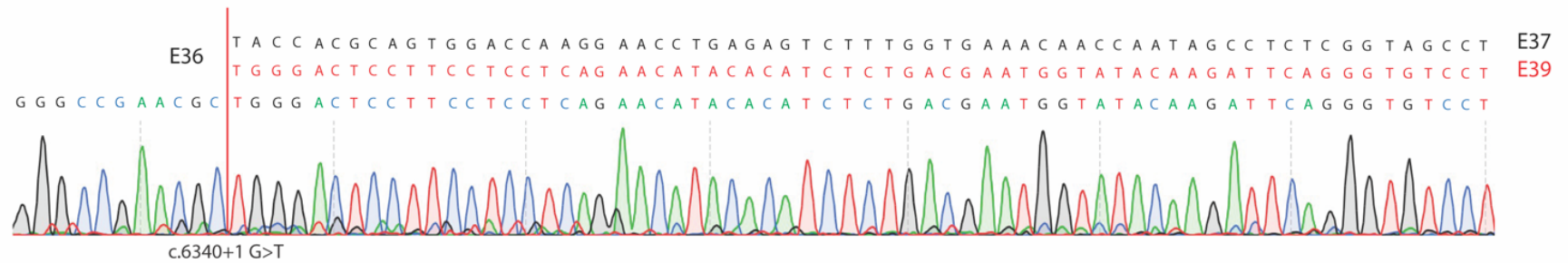

Supplement: Supplementary file 1 — Figure S1. [file ACN3-12-602-s002.pdf]
