# Supplementary material for: Clinical characterization of Collagen XII‐related disease caused by biallelic COL12A1 variants
Source: Ann Clin Transl Neurol. 2025 Feb 9;12(3):602–14. doi: 10.1002/acn3.52225 (PMC11920742; doi:10.1002/acn3.52225)
Supplement: Supplementary file 2 — Appendix S1. [file ACN3-12-602-s001.docx]

**Supplementary Methods**

| **Family** | **Methodology** | **Testing site** |
| --- | --- | --- |
| 1 | Trio WES (Commercial) | Baylor Genetics, Houston, TX, USA |
| 2 | Singleton WGS (Commercial) | Rady Children’s Institute of Genomic Medicine, San Diego, CA, USA |
| 3 | Neuromuscular Congenital Myopathy Panel (Commercial) | Viapath Laboratory, London, UK |
| 4 | Neuromuscular Congenital Myopathy Panel (Commercial) | Viapath Laboratory, London, UK |
| 5 | Singleton CLIA Neuromuscular Panel (Commercial) | Invitae, San Francisco, CA, USA |
| 6 | Trio WES (Commercial) | GeneDx, Gaitherburg, MD, USA |
| 7 | Singleton WES (Research) | Broad Institute of MIT and Harvard, Cambridge, MA, USA |

Supplementary Table 1. Genetic testing methods.

WES = whole exome sequencing; WGS = whole genome sequencing; CLIA = Clinical Laboratory Improvement Amendments

Segregation testing was pursued in all families and was consistent with recessive biallelic inheritance.

Targeted *COL12A1* mutation analysis was further performed with cDNA prepared from dermal fibroblast, which were treated and untreated with 0.2 mg/ml cycloheximide (CHX) (Sigma, Saint Louis, Missouri) for 19 hours to suppress nonsense-mediated mRNA decay. RNA was isolated from patient skin fibroblast cultures following the Qiagen miRNeasy kit manufacturer's protocol.Endpoint PCR was performed and followed by sequencing analysis.

**Supplementary Results**

*Clinical summaries*

**Family 1: P1** is a now 15-month-old girl of European descent. Pregnancy was notable for reduced fetal movement and positive Group B Streptococcus managed with antibiotics at time of delivery. She was born full term by Cesarean section following breech presentation, with arthrogryposis and micrognathia noted at delivery. She was noted to have abnormal posturing of the extremities, including her arms and legs, as well as bilateral finger flexor, wrist, and hand contractures. She was transferred to the Neonatal Intensive Care Unit (NICU) shortly after birth due to respiratory distress, transient hypoglycemia, and feeding difficulties ultimately requiring gastrostomy tube placement.

She had minimal movement in the first six months of life but subsequently gained the ability to lift her arms and legs against gravity. Acquisition of motor milestones was significantly delayed. She could roll to one side and bring her hands to her mouth at age six months and gained limited head control at age 13 months. She started babbling around the age of 6-7 months but has remained non-verbal at the age of 15 months. She had progressive kyphosis, along with persistent severe hypotonia and joint laxity, that remained unchanged from her neonatal presentation.

On examination she was found to have dolichocephaly, a long forehead with bi-temporal narrowing, and myopathic facies. She was found to have mild dysmorphic features including up-slanting palpebral fissures, almond shaped eyes with bluish-colored sclera, low set ears, micrognathia, and epicanthal folds. She had a high-arched palate with a deep midline groove and dental eruption cysts. She had severe head lag and had difficulty moving her head side to side when in a prone position at age 14 months.

Observational strength examination revealed her ability to move her upper and lower extremities against gravity. She was unable to raise her arms above her head. She was found to have contractures of the long finger flexors, fingers, and mild contractures of the toes.

Joint laxity was present in the shoulders, elbows, wrists, distal fingers, knees, ankles and toes. She had diffusely reduced muscle bulk throughout. She was found to have severe and generalized, appendicular > axial, hypotonia. Reflexes were trace or absent.

Serum creatinine kinase (CK) level was normal at 69 U/L. Muscle ultrasound demonstrated increased echogenicity throughout with notable muscle atrophy. Muscle MRI of the lower extremities demonstrated atrophy and diffuse fatty infiltration with patchy regions of STIR hyperintensity with bilateral symmetric involvement, albeit proximal more than distal muscles. Echocardiogram showed thickened mitral valve leaflets and mild-to-moderate mitral regurgitation. Brain MRI at age one month revealed some prominent CSF spaces around the whole brain and cerebellum, which was deemed within the range of normal. Previous chromosomal microarray testing identified a 28KB interstitial deletion of 14q31.3- q31.3 of unknown clinical significance.

**Family 2: P2** is a now 18-year-old girl of Palestinian descent. Family history was significant for consanguinity. Pregnancy was complicated by decreased fetal movements. At birth, she was noted to have arthrogryposis, hypotonia, hip dysplasia, weakness and respiratory insufficiency. She required a one-month NICU hospitalization for feeding difficulties ultimately requiring gastrostomy tube placement. Early on, she was found to have hypertrophic cardiomyopathy with left ventricular outflow tract obstruction and mitral valve prolapse which has reportedly improved over time.

Overall, gross and fine motor milestones were significantly delayed. She was able to sit supported at age three years. She showed slow developmental improvement and never experienced regression, although she never reached the ability to stand or ambulate independently. She had a history of speech delay, which has since resolved. Cognition was normal. She had progressive thoracolumbar scoliosis requiring surgery at age 10 years.

Examination at age 13 years revealed dolichocephaly, a long narrow face, micrognathia, high arched palate, dental malocclusion, and gingival hypertrophy. She had webbing of the neck, fingers, and knees. She had contractures of the knee and long finger flexors, and hyperlaxity of the thumb adduction, fingers and toes, with marked Achilles hyperlaxity with excessive dorsiflexion. Her skin was soft, she had palmar creases, mild thenar atrophy, and short lower limbs compared to her upper extremities. She had prominent calcanei, foot deformity, and pectus excavatum. Strength examination revealed proximal (MRC 5-/5) > distal (MRC 4-/5) weakness with significant deltoid and neck flexion involvement (MRC 2/5).

Serum CK was elevated in the 498-857 U/L range. EMG/NCS at age 4 years was reportedly normal. Muscle biopsy from the vastus lateralis at age seven years revealed fiber size variation and type I fiber predominance consistent with a myopathic process. Electron microscopy was normal. Brain MRI and EEG at age one month was normal. Echocardiogram at age 10 years revealed right ventricle restriction and left diastolic physiology in the absence of left ventricular hypertrophy. In addition to the *COL12A1* variants, WGS identified two variants of unknown significance including a maternally inherited c.8G>C; p.Cys3Ser missense variant in *LAMP2* and a paternally inherited c.671C>T p.Ala224Val missense variant in *CACNB2*.

**Family 3: P3** is a 4-year-old male of Afghani descent who was evaluated at the University of London College Hospital at age nine months old. Family history was significant for consanguinity. Pregnancy was complicated by reduced fetal movements. At birth he was noted to have congenital hypotonia, distal arthrogryposis, congenital hip dysplasia, and micrognathia. He had neonatal feeding difficulties with severe dysphagia ultimately requiring G-tube placement. Noninvasive nighttime ventilation was initiated. Gross and fine motor milestones were delayed and at age four years, he had limited head control and could not roll over or sit. On examination he was found to have micrognathia, high arched palate, gingival hypertrophy, and myopathic facies. He had a weak cry and a weak cough. He had finger contractures and kyphosis. Cardiac evaluation revealed dysplastic mitral and tricuspid valve with redundant tissue, mild mitral regurgitation, and a small patent ductus arteriosus.

**Family 4: P4** is a now 13-year-old Pakistani male. Pregnancy was notable for decreased fetal movements. At birth he was noted to have hypotonia and bilateral hip dislocation. He had neonatal feeding difficulties with dysphagia. Family history was significant for consanguinity. He also had a sister with congenital myotonia caused by homozygous pathogenic variant in *CLCN1* (c.1696G>A p.(Ala566Thr)). F4P4 was found to be heterozygous for this variant. His motor milestones were delayed. He was able to sit without support at age one year and walked independently at age two years. There has been no developmental regression or decline in strength. Cognitive development was normal. Examination revealed plagiocephaly, high arched palate, soft skin, overlapping fingers, and distal laxity. Muscle bulk was reduced in the lower extremities. Neuromuscular examination at age 13 years revealed mild proximal and axial weakness. Tone was normal. Forced Vital Capacity was 65% predicted at age 13 years. Muscle biopsy revealed mild variation in fiber size, increase in internal nuclei and a mild increase in connective tissue.

**Family 5: P5** is a 3-year-old girl of European descent. Pregnancy was notable for decreased fetal movements detected through biophysical profiles due to maternal PCOS. She was born via Cesarean section due to breech presentation. She had low tone and a weak cry at birth. Thick broad gums, high arched palate and bilateral hip dysplasia were also noted. She had a poor suck and hypoglycemia requiring nasogastric tube placement. She required occupational and speech therapy to transition to soft and solid foods. At age four months she was in a Pavlik harness to correct her hip dysplasia. At that time, she started physical therapy. She had delayed acquisition of motor milestones and achieved independent ambulation at 15 months but continued to have loss of balance and frequent falls. She had a history of speech delay, which has since resolved.

Neuromuscular exam revealed decreased axial and appendicular tone with normal muscle bulk. Hypermobility was noted at wrists, fingers, hips and feet. She had a mild hip adduction contracture and flat feet with prominent calcanei. Her gait was described as a slightly exaggerated hip swing with rotation of her knees inward, with mild feet pronation. Serum CK levels were normal. Echocardiogram was normal with trivial mitral regurgitation. Brain MRI at nine months showed mildly prominent lateral ventricles. Muscle ultrasound revealed diffuse, homogenous grade 1 mixed pattern of increased echogenicity in upper and lower extremities, with greatest involvement of the hamstrings, and the rectus femoris to a lesser extent.

**P6** is a 15-month-old younger sibling of P5. Pregnancy was complicated by decreased fetal movements at week 30 again detected through biophysical profiles due to maternal PCOS. He was born at term via repeat Cesarean section for breech presentation. At birth, he had transient respiratory distress that did not require prolonged respiratory support. He was noted to have decreased tone, torticollis, bilateral hip clicks, thick broad gums, and bilaterally undescended testes. Initial evaluation for hypotonia began at two months with targeted genetic testing revealing the same compound heterozygous variant as his older sister (**P5**). In comparison to his sister, he had milder hypotonia and poor head control, and improved his strength over time while receiving physical, occupational, and speech therapies. Currently, he does not pull to stand, but is able to cruise since age 11 months. On examination, he has a high arched palate and broad hypertrophic gingivae. He has hypermobility of the fingers, wrists, ankles and hips, without contractures. He appeared to have decreased axial and appendicular tone with normal muscle bulk. Muscle ultrasound demonstrated the same diffuse homogenous, grade 1 mixed pattern of increased echogenicity in extremities, again with greatest involvement of the hamstrings, and to a lesser extent the rectus femoris.

**Family 6: P7** is a now 3-year-old boy of European descent. Pregnancy was complicated by prior history of infertility with a history of Letrozole use and atypical PCOS. Fetal activity was felt to be within normal limits. He was born at term via repeat Cesarean section, with Apgar scores of 1, 6, 7 and 7 at one, five, ten and fifteen minutes respectively. He was noted to have arthrogryposis with right club foot and abnormal lower extremity positioning at birth and was found to have skeletal abnormalities including left femur and tibia fractures, thin ribs, possible right ulna fracture, displaced mandible and possible hip dislocation. He also had a duplicated renal collection system. He had chronic respiratory failure in the setting of an anatomic upper airway obstruction requiring tracheostomy and chronic mechanical ventilation at age one week. Over time he showed slow improvement and with no regression of symptoms. He was decannulated at age two. G-tube placed at one month, which he continues to rely on for most feeds but does take some snacks by mouth.

On physical examination at three years, he was found to have plagiocephaly, tall forehead, and bitemporal narrowing with metopic prominence. He was also noted to have high nasal bridge with round tip, prominent palatine ridges, crowded teeth, retrognathia, and mild facial asymmetry. Additionally, he had joint laxity and hypotonia in all extremities. He was missing distal interphalangeal creases on digits 3-5 (bilaterally) with no thenar creases and aberrant palmar creases. Fingers were long and slender with camptodactyly. Bilateral flexion contractures were noted at the knees, as well as feet with broad halluces bilaterally.

He has not yet achieved independent ambulation and relies mainly on a wheelchair, though he does use a gait trainer at home and school. He can sit unsupported for up to 15 minutes but cannot prop himself up to a seated position. He is speaking in phrases with dysarthric speech secondary to muscle weakness, though receptive and expressive language, as well as cognition, are felt to be age appropriate.

**Family 7: P8** is a 16-year-old-boy of Pakistani descent who was noted to have generalized hypotonia at birth. His motor milestones were significantly delayed. He had no head control and was unable to sit or crawl at age 12 months. Examination at age 12 months was notable for generalized hypotonia and weakness. He was able to roll from a supine into a prone position and vice versa with difficulties. He maintained his legs in a frog-like position when lying flat. He was noted to have bilateral knee flexion contractures. In a supine position he was unable to lift his legs from the ground. He could not grab his toes but was able to move toys from one hand into the other hand. He had bilateral prominent calcanei. He was found to have prominent distal joint hyperlaxity. His cry was very shallow, and he showed a high arched palate. He was unable to tolerate oral food intake and was fed via nasogastric tube.

Overtime, he did not show any regression of skills. He never achieved independent ambulation. Examination at age 16 years revealed a slight myopathic face. He had control of head movement and trunk movement in forward flexion. He was able to raise both elbows to shoulder height. He had severe weakness in his fingers and feet. He was able to bring his hand to his face and can feed himself. He had pronounced kyphosis, pectus excavatum and right convex scoliosis in his thoracic spine which had been progressive. He had knee contractures and hypermobility at the fingers. He had soft skin. His speech was slightly slurred. FVC was 69% sitting and 55% in supine position at age 12 years. His highest serum CK level was 500 U/L.

| **Genomic location^a^** | **Nucleotide change^b^** | **Predicted amino acid change** | **Predicted effect** | **gnomAD AF** | **ACMG variant classification^1^** |
| --- | --- | --- | --- | --- | --- |
| 6:75133291 | c.5794+2T>A |  | Impaired splicing | 0.00001314 | Pathogenic |
| 6:75137562 | c.5269C>T | p.R1757* | Nonsense | Absent | Pathogenic |
| 6:75102004 | c.8464C>T | p.R2822* | Nonsense | 0.000002052 | Pathogenic |
| 6:75128295 | c.6340+1G>T |  | Impaired splicing | Absent | Likely pathogenic |
| 6:75188413 | c.946_947insA | p.V316Dfs*6 | Frameshift | Absent | Pathogenic |
| 6:75155776 | c.3329C>A | p.A1110D | Missense | Absent | VUS |
| 6:75148468 | c.4177del | p.I1393Ffs*11 | Frameshift | Absent | Pathogenic |
| 6:75133857 | c.5664+1G>A |  | Impaired splicing | Absent | Likely pathogenic |
| 6:75130174 | c.6127del | p.V2043* | Nonsense | Absent | Pathogenic |
| 6:75138447 | c.5230+1G>A |  | Impaired splicing | 0.000004959 | Likely pathogenic |

Supplementary Table 1. Identified *COL12A1* variants

a Genome reference GRCh38

b Transcript ID: NM_004370.6

gnomAD AF, Genome aggregate database allele frequency (v4.1.1); ACMG, American College of Medical Genetics and Genomics; VUS, variant of uncertain significance.

1. Richards S, Aziz N, Bale S, Bick D, Das S, Gastier-Foster J, et al. Standards and guidelines for the interpretation of sequence variants: a joint consensus recommendation of the American College of Medical Genetics and Genomics and the Association for Molecular Pathology. Genet Med. 2015;17(5):405-24.

**Supplementary figures**

Supplementary Figure 1. cDNA testing on fibroblast (A) cDNA testing for patient F1P1 revealed that *COL12A1* c.5794+2T>A impairs splicing, resulting in out-of-frame skipping of exon 34. (B) cDNA testing for patient F3P3 revealed that *COL12A1* c.6340+1G>T impairs splicing, resulting in in-frame skipping of exon 37 and exon 38.
